# Host-Parasite Co-Evolution in Real-Time: Changes in Honey Bee Resistance Mechanisms and Mite Reproductive Strategies

**DOI:** 10.3390/insects12020120

**Published:** 2021-01-29

**Authors:** Arrigo Moro, Tjeerd Blacquière, Delphine Panziera, Vincent Dietemann, Peter Neumann

**Affiliations:** 1 Institute of Bee Health, Vetsuisse Faculty, University of Bern, CH-3097 Bern, Switzerland; peter.neumann@vetsuisse.unibe.ch; 2 Agroscope, Swiss Bee Research Center, CH-3003 Bern, Switzerland; vincent.dietemann@agroscope.admin.ch; 3 Wageningen Plant Research, Wageningen University & Research, 6708PB-1 Wageningen, The Netherlands; tjeerd.blacquiere@wur.nl (T.B.); delphine.panziera@wur.nl (D.P.); 4 Department of Ecology and Evolution, University of Lausanne, CH-1015 Lausanne, Switzerland

**Keywords:** *Apis mellifera*, co-evolution, honey bee, host, parasite, *Varroa destructor*

## Abstract

**Simple Summary:**

Parasitic mites, *Varroa destructor*, are a major threat for Western honey bees, *Apis* *mellifera*, colonies globally. Nevertheless, some honey bee populations can survive infestations with this mite, probably due to behaviors that suppress parasite reproduction. However, possible changes in mites associated with these surviving bees and the potential variations of bee behavior over time are poorly understood. Here, we show that mites can change their reproduction when associated with surviving hosts and that the bee behaviors suppressing mite reproduction can vary over time. In a fully-crossed field experiment on Dutch surviving colonies (Amsterdam Water Dunes (AWD) selection), worker brood cell recapping and varroa sensitive hygiene (VSH) performed by bees and mite reproductive parameters were investigated. Neither recapping nor VSH were significantly expressed even though a previous study showed VSH in these AWD bees. A larger proportion of mites that co-evolved with AWD surviving bees reproduced compared to mites in routinely treated colonies, but had lower fecundity. Overall, our study suggests that honeybee colonies can survive infestations with these mites by not yet understood means and shows for the first time adaptive changes in the reproduction of their coevolved mites.

**Abstract:**

Co-evolution is a major driving force shaping the outcome of host-parasite interactions over time. After host shifts, the lack of co-evolution can have a drastic impact on novel host populations. Nevertheless, it is known that Western honey bee (*Apis* *mellifera*) populations can cope with host-shifted ectoparasitic mites (*Varroa destructor*) by means of natural selection. However, adaptive phenotypic traits of the parasites and temporal variations in host resistance behavior are poorly understood. Here, we show that mites made adaptive shifts in reproductive strategy when associated with resistant hosts and that host resistance traits can change over time. In a fully-crossed field experiment, worker brood cells of local adapted and non-adapted (control) *A.* *mellifera* host colonies were infested with mites originating from both types of host colonies. Then, mite reproduction as well as recapping of cells and removal of infested brood (i.e., Varroa Sensitive Hygiene, VSH) by host workers were investigated and compared to data from the same groups of host colonies three years earlier. The data suggest adaptive shifts in mite reproductive strategies, because mites from adapted hosts have higher probabilities of reproduction, but lower fecundity, when infesting their associated hosts than mites in treated colonies. The results confirm that adapted hosts can reduce mite reproductive success. However, neither recapping of cells nor VSH were significantly expressed, even though the latter was significantly expressed in this adapted population three years earlier. This suggests temporal variation in the expression of adaptive host traits. It also appears as if mechanisms not investigated here were responsible for the reduced mite reproduction in the adapted hosts. In conclusion, a holistic view including mite adaptations and studies of the same parasite/host populations over time appears overdue to finally understand the mechanisms enabling survival of *V.* *destructor*-infested honey bee host colonies.

## 1. Introduction

Coevolution is a dynamic process driving the interactions between parasites and hosts [1,2,3]. Such dynamics can vary across generations depending on specific selection scenarios [3]. As parasites are considered among the organisms with the highest evolutionary potential, hosts will have to swiftly evolve efficient adaptive strategies in order to survive [4]. The remarkable adaptability of parasites includes the propensity to switch to new hosts, for which the lack of coevolution can have disastrous consequences [5]. A representative example of this is *Varroa destructor*, an ectoparasitic mite, which is currently considered as the most devastating threat to the survival of its novel host, the Western honey bee, *Apis mellifera* [6]. *V. destructor* causes little harm to its original host, the Eastern honey bee, *Apis cerana*, since this species shares a long co-evolutionary history that led to the development of defense mechanisms [7,8,9,10]. However, when *V. destructor* switched to *A. mellifera* around the middle of the last century, its initial high virulence was not counteracted by co-evolved host defenses, which led to the quasi-eradication of wild and feral honey bee populations in the Northern hemisphere [11,12,13] and to high losses of managed colonies [14].

On the other hand, the evolution of resistance to mite infestations by means of natural selection has been demonstrated in several populations of Western honey bees, in which no chemical treatments against the mites were implemented (reviewed by [15]). All *A. mellifera* populations in sub-Saharan Africa as well as Africanized populations in the Americas survive without treatments, while those of European origin are in general susceptible [10,15]. Nevertheless, surviving European *A. mellifera* populations and populations of European-derived *A. mellifera* in North-America have been reported [15,16,17,18,19,20,21]. In these colonies, natural selection fostered the evolution of traits that enabled them to cope with the parasite. Some of these adaptations are specific behaviors of workers, which are targeting mite-infested brood cells, thereby reducing parasite reproductive success [21]. For example, in four European surviving *A. mellifera* populations, workers have evolved the capacity to detect brood cells containing reproductive mites and, by opening and closing the cell cap (i.e., cell recapping), may interfere with the reproduction of the parasite ([22], but see [23,24]). Resistant populations can also remove the entire content of brood cells infested with reproductive mites (i.e., varroa sensitive hygiene (VSH) [25] as in the case of the Amsterdam Water Dunes selection, AWD, studied by Panziera et al. [20]). These results demonstrate the remarkable capacity of Western honey bees to rapidly adapt to a novel parasite. However, the possibility that the parasite may also adapt as a response to the selective pressure imposed by these resistant hosts has so far received little attention [26].

*V. destructor* reproduces in capped brood cells of its hosts. A female foundress mite will first lay a haploid egg, which will develop into a male, followed by three to four diploid eggs, which will develop into females [27]. The male mates with its sisters and the reproductive cycle ends at the emergence of the host [6,10]. Due to this sib-mating system as well as initial findings of clonal lineages and high levels of inbreeding due to bottlenecks [28], *V. destructor* has been regarded as a parasite with low evolutionary potential [26]. Nevertheless, several studies demonstrated that *V. destructor* has ample capacity to adapt under high selective pressures, e.g., several populations of mites treated with synthetic acaricides rapidly evolved resistance [29,30,31,32]. Population genetics data also suggest adaptive changes in mites coevolving with surviving hosts [33]. However, the phenotypic traits of *V. destructor* enabling it to cope with adapted hosts are poorly understood. The established co-evolved system of the original host Eastern honey bees *Apis cerana* and *Varroa* sp. mites [7,9,10] might enable the prediction of adaptive phenotypic traits of *V. destructor* to naturally surviving new *A. mellifera* hosts. In *A. cerana*, *Varroa* sp. mites reproduce with rare exceptions only in seasonally occurring males (i.e., drone) brood [9], possibly to avoid worker adult bee and/or brood defense mechanisms [8]. Assuming that the selective pressure imposed by surviving *A. mellifera* is similar to those imposed by the original host *A. cerana*, we would expect a reduced virulence (parasite-induced colony mortality, [19]), possibly driven by adaptations towards lower rates of reproduction in mother mites (i.e., lower fecundity). Since a lower mite fecundity may be less likely to trigger host-resistance responses [34], a higher fertility (i.e., probability of reproduction) combined with lower fecundity is likely to ultimately enhance the fitness of the parasites. Adaptations of these mites have been suggested earlier [19,35], but have not yet been demonstrated empirically.

Spatiotemporal variation of both host and parasite traits is key to our understanding of host parasite co-evolution [1,2,3,36]. As such, there is a need to integrate a temporal perspective whenever investigating mechanisms of resistance enabling hosts to survive, especially in relation to recently shifted parasites [5,37]. Unfortunately, for surviving Varroa-infested Western honey bees, data on year to year fluctuation in the expression of resistance traits are scarce [38]. As such, it appears overdue to take advantage of a holistic coevolutionary approach considering key adaptive phenotypic traits in both host and parasite [26] and simultaneously evaluate their changes across the temporal scale [37]. This can be achieved by repeating at multiple points in time fully-crossed experiments involving honey bees and mites, which coevolved under different selection scenarios. Here, we considered the case of the Dutch bees from the AWD selection [20,39,40,41]. This is a population selected for survival in the absence of acaricide treatments following a Darwinian black box beekeeping protocol [42]. To date, this selected lineage has been surviving without acaricide treatments for 12 years and is extensively used in beekeeping exploitations (T. Blacquière, unpublished). For the parasite side, we investigated the overall reproductive success as a measure of virulence as well as the success rate of initiated reproduction as a token of the parasite’s ability to overcome host defense. We measured these parameters in mites infesting the AWD honey bee lineage and in mites infesting regularly treated and unselected local control colonies. Mites of each type were introduced in both selected and unselected colonies, which originated from the same population and thus had the same genetic background. As investigations on the reproductive capacity of experimentally introduced mites had already been done in a previous study on the same selected and treated host populations [20], it was possible to consider changes in reproductive success and host defense mechanisms over time. To investigate mite reproduction and the resistance mechanisms of the host honeybees, we used exactly the same protocols to record VSH [25] as in Panziera et al. [20] and also included brood cell recapping [22]. We then compared the expression of resistance mechanisms with that of the same groups of treated unselected and local colonies as in Panziera et al. [20] to investigate possible changes in host traits over time. In light of previous studies [20,22], we expected the bees from the selected lineage to express brood cell recapping and VSH more readily than the treated ones. However, given that the investigated traits may change over time they may be more or less readily expressed pending the selection pressure. Finally, we expected the mites from selected host colonies to have, analogous to the original host *A. cerana*, a reduced fecundity (i.e., individual reproductive output), but nevertheless having a higher probability of reproducing (i.e., fertility) compared to mites from treated colonies.

## 2. Materials and Methods

### 2.1. Experimental Setup

In summer 2018, two groups of honey bee colonies originating from the very same populations tested in Panziera et al. [20], one originated by following a Darwinian black box selection protocol [42], and composed of colonies from the AWD selection (*N* = 5, termed “selected” hereafter) and a second one originated through a conventional local beekeeping approach (*N* = 6, “treated” hereafter) were set up in an experimental apiary in Wageningen, The Netherlands. Notably, the study was conducted in the same period of the year as in Panziera et al. [20]. To minimize the impact of different genotype–environment interactions [43], the treated group was formed from local susceptible colonies, which were regularly treated against *V. destructor* infestations [44]. Lastly, starting from three weeks before the experiment, two additional colonies from each group were selected as mite sources [20]. These colonies were managed in order to foster the production of mites. In one colony of each pair, all frames with brood cells that would have been sealed within the next six to eight hours (i.e., cells containing mature larvae about to pupate) were removed and placed in the paired one. In parallel, from this second colony, combs with emerging workers were extracted and transferred to the first one. As a result, for each pair, one colony mostly contained mites infesting capped brood cells, while the other had mostly mites residing on adult bees. Mites used for this experiment were sampled from the latter using the icing sugar method [45].

### 2.2. Experimental Infestations

Brood frames were briefly extracted from the colonies to map worker brood cells ready to be capped using transparent acetate sheet, and then returned to their colony of origin [20,45]. In parallel, adult female *V. destructor* mites were collected from the source colonies using the icing sugar method [45] and kept in plastic containers with a moist tissue until their introduction in brood cells.

Six hours after cell marking, the mapped combs were extracted from each colony and freshly capped worker brood cells were identified using the transparent sheets (*N* = 30 for each colony). On these cells, experimental infestations were carried out by making a small incision at the side of each cell cap, introducing a single live mite with a fine paintbrush and then carefully closing the incision by pushing the wax cap down [45]. The experimentally infested cells were divided in four treatment groups depending on the combination of mites and bee hosts: selected mites infesting the selected host lineage introduced back into brood cells of this lineage (= selected-selected), mites infesting treated colonies introduced in brood cells of the selected lineage (= treated-selected), mites infesting the selected host lineage introduced in brood cells of treated colonies (= selected-treated) and mites infesting the treated colonies back into brood cells of the treated colonies (= treated-treated).

### 2.3. Assessment of Cell Recapping, Varroa Sensitive Hygiene and Mite Reproduction

The brood frames were removed from the colonies ten days after experimental infestation, a time when the introduced female mites should have laid all eggs and the male offspring should be mature and hence when reproductive success can be measured [45]. Only cells that were found to be infested by a single mother mite at the moment of inspection were considered for the analyses. This was in order to account for the possibility that, during the few hours in which mapped combs were returned to the colonies for cell capping, cells may have been naturally infested by mites present in the colonies [45]. To assess cell recapping rates, each cell was opened and the underside of the capping was inspected [46]. If the glossy layer of silk cocoon was lacking and a wax plug visible, the cell was considered as being recapped. If instead the silk layer was intact, the cell was considered as being non-recapped. Additionally, varroa sensitive hygiene (VSH, [25]) was inferred by determining the rates of brood removal derived from the number of experimentally infested cells, which were found empty at the moment of inspection [20].

For each cell, the pupae were carefully extracted to analyze the mite family composition as well as the reproductive success of each inserted foundress mite [45,47]. Foundress mites were categorized as successfully reproductive, if they produced one sexually mature male and at least one sexually mature daughter [45]. If the male mite was not present, or if a male was not accompanied by at least one sexually mature daughter, or if no offspring were produced, the foundress mites were considered as non-reproductive. Lastly, for each reproductive female mite, fecundity was determined by counting the number of daughters produced.

### 2.4. Statistical Analyses

The data were analyzed using the statistical software *R* [48]. In order to estimate the probabilities of brood cell recapping, brood cell removal, and successful mite reproduction in the four groups, mixed-effect logistic regression models were implemented using the package *lme4* [49]. Each cell was considered as an independent sampling unit. Cell recapping, brood cell removal, and successful mite reproduction were considered as response factors with binomial distribution (1 in case the event did happen, 0 in case the event did not happen). Treatment group was considered as a fixed explanatory variable and colony identity was considered as a random one. In addition, to allow for comparisons over time (three years; data collection Panziera et al. [20]: 2015; this data set: 2018), the estimated probabilities of mite reproduction and brood cell removal obtained for treated-associated mites infesting selected and treated bees were compared with those reported in Panziera et al. [20]. For this, mixed-effect logistic regression models were run by considering mite reproduction or brood removal as response variable, with year as a fixed effect and colony identity as a random one.

To compare the selected parameters between groups considered in each model, adjusted mean proportions and pairwise comparisons (Tukey HSD method) were calculated using the emmeans package [50]. Lastly, as the sample size did not allow for an ideal convergence of a fully designed model, the average number of daughters produced by foundress mites (i.e., fecundity) in the four groups were compared using Kruskall-Wallis tests, followed by pairwise Wilcoxon tests coupled with Bonferroni adjustments for allowing correction after multiple comparisons.

## 3. Results

**Figure 1:**
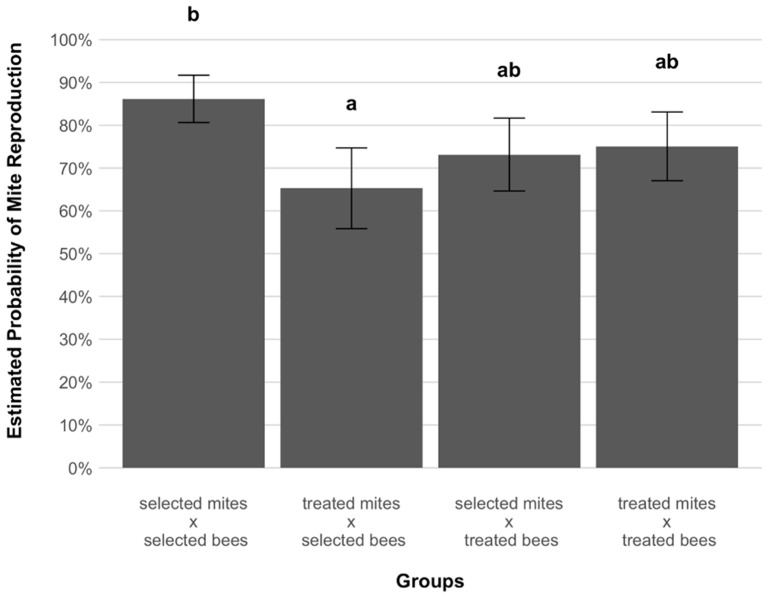
Estimated probabilities of reproduction (±SE) of female mites, *Varroa destructor*, in experimentally infested worker brood cells of the different honey bee, *Apis mellifera*, host groups (selected-selected, treated-selected, selected-treated, treated-treated). After adjustment for multiple comparison (Tukey method), significant differences were detected between groups as indicated with letters above the bars (GLMM: χ^2^ = 9.832, *p* = 0.02). Both the highest and the lowest reproductive probabilities were detected in mites infesting AWD surviving bees, where selected-associated mites showed a statistically higher reproductive capacity than treated-associated one.

**Figure 2:**
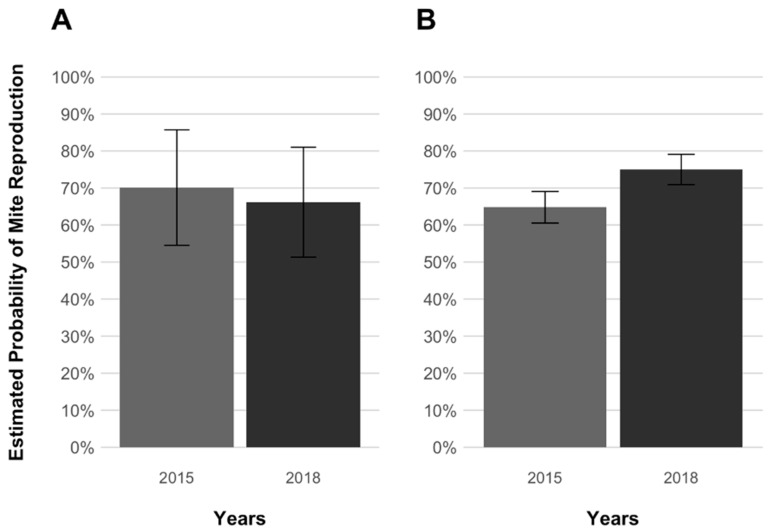
Temporal comparison of the estimated probabilities of reproduction (±SE) of female mites, *Varroa destructor*, from treated colonies in experimentally infested worker brood cells of *Apis mellifera* selected (**A**) and treated (**B**) colony groups. Earlier data (2015) from Panziera et al. [20] are in light grey and present data (2018) in dark grey. The earlier data refer to the groups AWD (treated-selected) and C (treated-treated) as reported in Panziera et al. [20]. No significant differences were detected between the estimated probabilities.

Overall, a total of 507 cells were singly infested by live foundresses and used for analyses (Table 1). After inspection, in the cells from which the brood was not removed by adult workers, 302 foundresses (67.7%) were found to be reproductive and 113 (25.3%) to be non-reproductive (Table 2). Interestingly, the highest and the lowest reproduction probabilities were found when mites infested brood of the selected lineage (Figure 1): a significantly higher probability of reproduction was found for mites from the selected lineage compared to mites from the treated colonies (GLMM, *p* = 0.009, Figure 1). In contrast, no significant difference in reproductive probabilities was observed between the two groups of mites when infesting the brood of treated colonies (GLMM, *p* = 0.98, Figure 1). Moreover, when the estimated probabilities of reproduction of treated-associated mites infesting selected and treated colonies were compared with those reported in Panziera et al. [20], no significant differences were found (Table S1, Figure 2).

**Figure 3:**
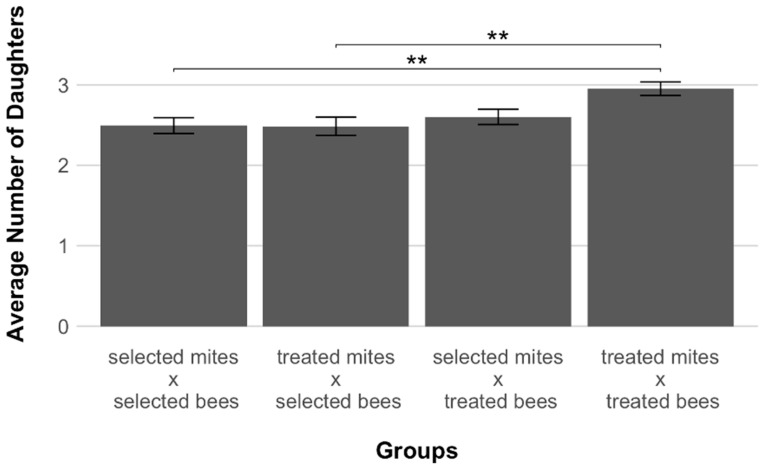
Average number of daughters (±SE) produced by female mites, *Varroa destructor*, in experimentally infested honey bee, *Apis mellifera*, worker brood cells of the different host groups (selected-selected, treated-selected, selected-treated, treated-treated). After Bonferroni adjustment, significant differences were detected between groups (** = *p* < 0.01). Both selected and treated mites infesting selected bees produced significantly less daughters than treated-associated mites infesting their original host.

The average number of daughters produced by successfully reproducing foundress mites ranged from a minimum of 2.48 (±0.11 SE) for treated-associated mites infesting brood cells of selected bees, to a maximum of 2.95 (±0.08 SE) for treated-associated mites infesting brood cells of their original host (Figure 3). A statistically significant difference in fecundity was found when the groups were compared (Kruskal–Wallis test, *p* = 0.005, Figure 3). The two groups of mites infesting the selected lineage produced significantly less daughters than the group of treated-associated mites infesting their original host (Bonferroni adjusted *p* = 0.006 and 0.009, Figure 3).

**Figure 4:**
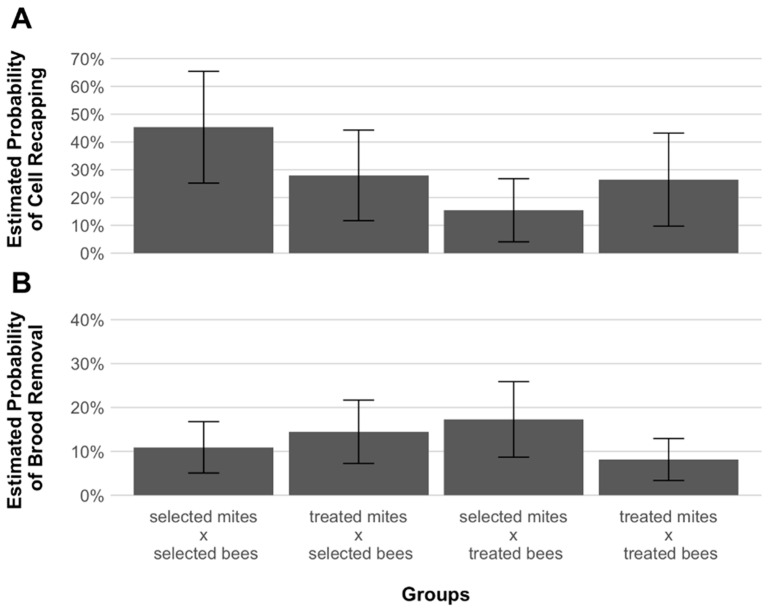
Estimated probabilities of honey bee, *Apis mellifera*, worker brood cell recapping of (**A**) and brood removal in (**B**) mite, *Varroa destructor*, experimentally infested cells in the different host groups (selected-selected, treated-selected, selected-treated, treated-treated). Error bars indicate standard error of the mean. There were no significant differences between the tested groups for either cell recapping (GLMM: χ^2^ = 6.991, *p* = 0.072) or brood removal (GLMM: χ^2^ = 5.763, *p* = 0.123).

Among the infested brood cells, 129 (25.4%) were found to be recapped, the content of 92 (18.1%) had been removed and 286 (56.4%) were not manipulated by the adult bees (Table 1). The estimated probabilities of cell recapping ranged from a maximum of 45.3% for selected-associated mites infesting cells of their hosts, to a minimum of 15.4% for treated-associated mites infesting cells of selected hosts (Figure 4). No significant difference in the estimated probabilities of cell recapping between the groups was detected (*p* = 0.072, Table S2). Likewise, no significant effect of cell recapping over mite reproduction was found (*p* = 0.955, Table S3). Lastly, both the highest and the lowest levels of brood cell removal probabilities were found in cells of treated bees (17.3% for selected-associated mites and to 8% for treated-associated ones, Figure 4). There was no significant difference in the estimated probabilities of brood removal between the different groups (*p* = 0.123, Table S4, Figure 4).

**Figure 5:**
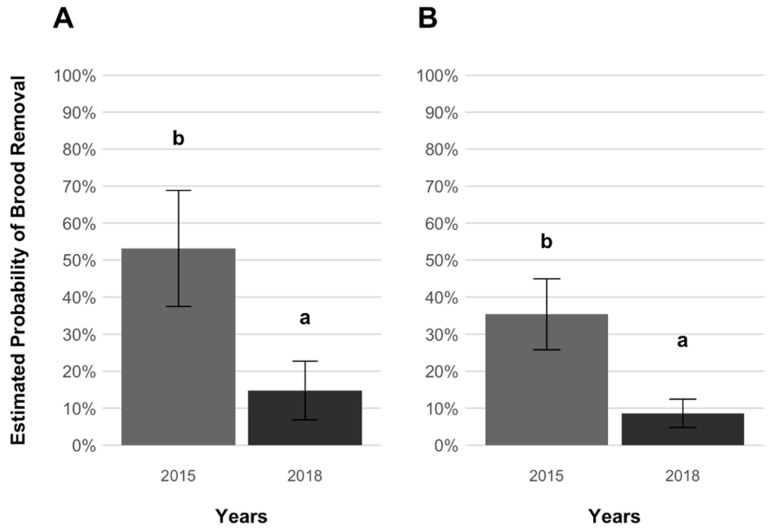
Temporal comparison of the estimated probabilities of worker brood removal (±SE) in selected (**A**) and treated (**B**) groups of *Apis mellifera* cells experimentally infested with *Varroa destructor* mites associated with treated colonies. Earlier data (2015; Panziera et al. [20]) are indicated in light grey and present data (2018) in dark grey. The earlier data refer to the groups AWD (treated-selected) and C (treated-treated) as reported in Panziera et al. [20]. Significant differences were detected when comparing the probabilities of brood removal across time for both host groups as indicated by different letters (*p* = 0.035 and *p* = 0.006, respectively).

Lastly, in the comparison between present (2018) and earlier (2015) brood removal probabilities obtained for cells infested with treated-associated mites, a significantly lower expression of brood removal was found between the present data and those of Panziera et al. [20] for both selected and treated bees within three years (GLMM, *p* = 0.035 and *p* = 0.006, Table S5, Figure 5).

## 4. Discussion

The data show clear evidence for the adaptive potential of both host and parasite as predicted by co-evolutionary theory [3]. Ectoparasitic mites *V. destructor* coevolved with selected hosts presented a different reproductive strategy when compared to non-coevolved ones. Moreover, surviving bees apparently changed their resistance mechanisms within a time window of three years. Mites coevolved with selected bees had significantly higher probabilities of successful reproduction in their associated hosts compared to mites from treated colonies. In addition, the average number of daughters produced by foundress mites from selected bees infesting their hosts was significantly lower compared to that of mites associated with treated hosts on their host, suggesting a reduced virulence in the former. Moreover, foundress mites from treated hosts produced significantly less daughters in AWD selected bees compared to the treated hosts, thereby confirming that the selected AWD host lineage has evolved means to suppress mite reproduction. However, neither varroa sensitive hygienic behavior nor recapping can explain this suppression, suggesting that AWD bees reduce mite reproductive success by yet unknown mechanisms.

Previous studies on the surviving colonies of the AWD selection only used mites from treated colonies [20]. For this same group of mites, the present data are in line with the earlier estimated probabilities of successful reproduction (Table S1, Figure 2), suggesting that no changes in the reproductive capacities of treated-associated mites have taken place since the previous study. This supports the idea that mites do not have to adapt to the host because of colony treatments [51]. This comparison is not available for mites associated with bees of the selected lineage. However, when considering the two mite groups infesting AWD bees in 2018, a differential success of reproduction was detected as mites not co-evolving with AWD bees had a significantly lower probability of successful reproduction compared to mites co-evolving with this host (65.3% vs. 86.2%, *p* = 0.009, Figure 1). As this differential success was not observed in the two mite groups infesting treated host groups (73.2% vs. 75.1%, *p* = 0.98, Figure 1), and given that this high fertility is comparable to that expected for mites infesting non-adapted hosts [10], co-evolution may have fostered the development of adaptive reproductive strategies in mites infesting selected colonies that enable them to reproduce successfully when infesting their resistant host.

This increase in the probability of reproduction was associated with a reduced number of daughters produced per foundress mite (Figure 3), which is in line with the prediction that mite variants with a reduced reproduction will be selected via coevolution leading to an equilibrium with the new host [19]. As the survival of *V. destructor* in the original host *A. cerana* is considered to be due to adaptations in reproductive strategy [9], it seems plausible that a reduced fecundity in adapted *A. mellifera* worker brood cells may also ultimately enhance mites’ fitness. Indeed, a high fertility coupled with low fecundity, i.e., fewer offspring, will leave the foundress more resources to invest in subsequent reproductive cycles. Since fewer mite offspring feeding on the brood will be less likely to trigger host responses, the investment of fewer eggs per reproductive cycle appears adaptive. Whether mites from AWD colonies actually trade immediate for long-term fitness (producing less offspring per reproductive cycle, but going through more cycles during their lifetime), could be determined in future studies by comparing the individual *V. destructor* lifetime reproductive success when associated with selected and treated *A. mellifera* host colonies. Lastly, given that selected-associated mites also showed a reduced fecundity in treated colonies and that treated-associated mites also produced a significantly lower number of daughters when infesting cells of selected bees in comparison to those of their associated host (Figure 3), it is unclear whether fecundity is determined by parasite or host mechanisms.

Many studies have suggested the capacity of mite-adapted honey bees to suppress mite reproduction by various mechanisms [21]. Recently, among these mechanisms, the recapping of infested brood cells has been consistently found in resistant populations [22,24,46]. Our data show that the probabilities of mite reproduction did not significantly differ based on whether a cell had been recapped or not (Table S1). Moreover, AWD colonies had recapping proportions comparable to those of treated colonies (Figure 4). It therefore appears as if the AWD selected colonies may not rely on targeted brood cell recapping to suppress mite reproduction, similar to other surviving honey bee populations [23].

When honey bee workers express varroa sensitive hygiene behavior, they uncap infested cells and remove the brood they contain [25]. In this way, the reproduction of the mite is inevitably disrupted. The results of the present study show that selected and treated colonies did not differ in VSH when infested with treated mites (Figure 4). It thus seems safe to conclude that the confirmed reduced reproductive success of mites in the AWD bees can also not be explained by the removal of infested brood. Instead, other traits of resistance must explain the observed patterns of mite infestations, e.g., drone pupae of AWD bees seem to interfere with mite oogenesis [39]. A reduced mite oogenesis may also occur in infested worker brood because our data show that the number of offspring produced by both mite groups infesting selected bees was significantly lower than that of treated-associated mites infesting their hosts (Figure 3).

The earlier data [20] suggested that in 2015, AWD bees relied on varroa sensitive hygiene to suppress mite reproduction. However, the results of the present study show that the AWD bees removed significantly less infested brood cells in 2018 compared to 2015. Since non-removed pupae in VSH colonies are able to suppress mite reproduction [52] and worker and drone pupae of AWD bees may interfere with mite oogenesis ([39]; our data), less costly brood resistance traits might have been favored [53,54]. In honey bee populations in which the removal of infested brood appears to be a major trait of resistance, its expression is known to be dependent on seasonal conditions and the availability of environmental resources (i.e., nectar) [38,55], as well as the proportion of infested brood cells [56], which results in the need to perform multiple measurements to reliably assess VSH expression [57]. However, these factors seem unlikely to be decisive since the present and the earlier study [20] have both been conducted in very similar experimental settings (i.e., same season and location with similar nectar availability) and equivalent levels of mite infestation (T. Blacquière, unpublished data). Nevertheless, environmental factors cannot be excluded completely because the level of VSH in control colonies, which are less subjected to selective pressure by the parasite, also decreased between 2015 and 2018. Finally, epigenetics may be involved, i.e., heritable changes in bee behavior that do not involve alterations in the DNA sequence [58]. Irrespective of whether the decrease in VSH in the selected colonies is due to genetics or environmental factors, an apparent change in the resistance mechanisms used against *V. destructor* has occurred.

## 5. Conclusions

Evidence of the capacity of mites *V. destructor* to adapt to *A. mellifera* hosts was lacking. Here, the results of a fully-crossed field experiment indicate that mites associated with surviving AWD bees adapted their reproductive strategy to this host as to possibly counteract yet unidentified traits of resistance impeding mite fecundity. The recapping of brood cells had no significant impact in the present study and the selected colonies were less frequently expressing brood removal compared to three years earlier, suggesting the importance of other as yet unidentified mechanisms. These results also suggest a shift in host resistance mechanisms within a three-year period. Although the mechanistic explanations of how mites and bees are adapting to each other are currently lacking, these results confirm the dynamic nature of coevolution [3,37] and call for a holistic view of this particular host-parasite system, including investigations of mite and bee traits in the very same populations over time. Such an approach is required to finally enable a more complete mechanistic understanding of the ability of *A. mellifera* colonies to survive infestations by *V. destructor* by means of natural selection. This may also offer an avenue towards a more sustainable beekeeping with *A. mellifera* globally.

## Figures and Tables

**Table 1:** Fully-crossed infestation field experiment with mites, *Varroa destructor*, and worker brood cells from both selected and treated honey bee, *Apis mellifera*, colonies. The total number of cells experimentally infested with foundress mites and the numbers and proportions (%) of cell recapping, brood removal and untouched cells are shown for the four groups.

Group	Cells			
	Total	Recapped	Content Removed	Untouched
	*N*	%	%	%
Selected mites × selected honey bees	115	29.6%	17.4%	53.0%
Treated mites × selected honey bees	135	23.0%	25.2%	51.9%
Selected mites × treated honey bees	133	18.0%	19.5%	62.4%
Treated mites × treated honey bees	124	32.3%	9.7%	58.1%
Total	507	25.4%	18.1%	56.4%

**Table 2:** Reproductive status of female foundress mites, *Varroa destructor*, from selected or treated honey bee, *Apis mellifera*, host colonies in association with experimentally infested worker brood cells of either selected or treated host colonies in the fully-crossed field experiment (selected-selected, treated-selected, selected-treated, treated-treated, respectively). The total numbers of mites as well as the number (*N*) and proportions (%) of reproductive and non-reproductive mites are shown.

Group	Total	Reproductive Mites	Non-Reproductive Mites
	*N*	%	%
Selected mites × selected honey bees	95	82.1%	17.9%
Treated mites × selected honey bees	101	61.4%	38.6%
Selected mites × treated honey bees	107	72.9%	27.1%
Treated mites × treated honey bees	112	75.0%	25.0%
Total	415	72.8%	27.2%

## Data Availability

The data presented in this study are available from the corresponding author after reasonable request.

## Supplementary Materials

The following are available online at https://www.mdpi.com/2075-4450/12/2/120/s1, Table S1: Logistic regression model used for estimating the probabilities of reproduction of female mites, *Varroa destructor*, associated with treated colonies in experimentally infested worker brood cells of selected and treated *Apis mellifera* colonies from the present (2018) and the earlier data (2015, [20]). Table S2: Fixed explanatory variables used in the logistic regression model for estimating the probabilities of honey bee, *Apis mellifera*, worker brood cell recapping in the experimentally infested cells of the four groups of female mites, *Varroa* destructor, in the fully-crossed experimental infestation experiment (selected-selected, treated-selected, selected-treated, treated-treated). Table S3: Fixed explanatory variables used in the logistic regression model implemented for estimating the probabilities of successful mite, Varroa destructor, reproduction in experimentally infested honey bee worker brood cells, *Apis mellifera*. Table S4: Logistic regression model used to estimate the probabilities of honey bee, *Apis mellifera*, worker brood removal in the experimentally infested cells for the four groups of female mites (selected-selected, treated-selected, selected-treated, treated-treated). Table S5: Logistic regression models used for estimating the probabilities of hygienic brood removal in experimentally infested worker brood cells of *Apis mellifera* selected and treated colonies from the present and the earlier study [20].
