# Supplementary material for: Host-Parasite Co-Evolution in Real-Time: Changes in Honey Bee Resistance Mechanisms and Mite Reproductive Strategies
Source: Insects. 2021 Jan 29;12(2):120. doi: 10.3390/insects12020120 (PMC7911685; doi:10.3390/insects12020120)
Supplement: Supplementary file 1 [file insects-12-00120-s001.pdf]

Table S1. Logistic regression model used for estimating the probabilities of reproduction of female mites, *Varroa destructor*, associated with treated colonies in experimentally infested worker brood cells of selected and treated *Apis mellifera* colonies from the present (2018) and the earlier data (2015, [1]). Each experimentally infested cell has been considered as a single statistical unit. Mite reproduction was considered as a binomial variable (1 for successful mite reproduction, 0 for the opposite case). Each cell group was used as fixed explanatory variable while colony identity was used as a random one.

Temporal Comparison	Response Variable	Explanatory Variable	Degrees of Freedom	$\chi 2$	*p*-value
Treated mites × selected bees	Mite Reproduction	Year	1	0.033	0.855
Treated mites × treated bees	Mite Reproduction	Year	1	2.884	0.089

Table S2. Fixed explanatory variables used in the logistic regression model for estimating the probabilities of honey bee, *Apis mellifera*, worker brood cell recapping in the experimentally infested cells of the four groups of female mites, *Varroa destructor*, in the fully-crossed experimental infestation experiment (selected-selected, treated-selected, selected-treated, treated-treated).

Response Variable	Explanatory Variable	Degrees of Freedom	$\chi 2$	*p*-value
Cell Recapping	Groups	3	6.991	0.072
	Mite Reproduction	1	0.032	0.857

Table S3: Fixed explanatory variables used in the logistic regression model implemented for estimating the probabilities of successful mite, *Varroa destructor*, reproduction in experimentally infested honey bee worker brood cells, *Apis mellifera*.

Response Variable	Explanatory Variable	Degrees of Freedom	$\chi 2$	*p*-value
Mite Reproduction	Groups	3	9.832	0.0200
	Cell Recapping	1	0.003	0.955

Table S4: Logistic regression model used to estimate the probabilities of honey bee, *Apis mellifera*, worker brood removal in the experimentally infested cells for the four groups of female mites (selected-selected, treated-selected, selected-treated, treated-treated).

Response Variable	Explanatory Variable	Degrees of Freedom	$\chi 2$	*p*-value
Brood Removal	Groups	3	5.763	0.123

Table S5: Logistic regression models used for estimating the probabilities of hygienic brood removal in experimentally infested worker brood cells of *Apis mellifera* selected and treated colonies from the present and the earlier study [1].

Temporal Comparison	Response Variable	Explanatory Variable	Degrees of Freedom	$\chi 2$	*p*-value
Treated mites × selected bees	Brood Removal	Year	1	4.424	0.035
Treated mites and treated bees	Brood Removal	Year	1	7.483	0.006

Reference

1. Panziera, D.; van Langevelde, F.; Blacquière, T. Varroa sensitive hygiene contributes to naturally selected varroa resistance in honey bees. *J. Apic. Res.* 2017, *56*, 635–642, doi:10.1080/00218839.2017.1351860.
